# Prognostic analysis of postoperative clinically nonmetastatic renal cell carcinoma

**DOI:** 10.1002/cam4.2775

**Published:** 2019-12-16

**Authors:** Yanxiang Shao, Sanchao Xiong, Guangxi Sun, Weichao Dou, Xu Hu, Weixiao Yang, Thongher Lia, Shi Deng, Qiang Wei, Hao Zeng, Xiang Li

**Affiliations:** ^1^ Department of Urology Institute of Urology West China Hospital Sichuan University Chengdu China

**Keywords:** carcinoma, renal cell, prediction model, prognostic factor, survival

## Abstract

**Objectives:**

To investigate the survival characteristics of postoperative nonmetastatic renal cell carcinoma (RCC) patients, and the predictive value of a prognostic model.

**Materials and Methods:**

We retrospectively evaluated data from 1202 postoperative nonmetastatic RCC patients who were treated between 1999 and 2012 at West China Hospital, Sichuan University (Chengdu, China). In addition, we also evaluated data relating to 53 205 cases acquired from the Surveillance, Epidemiology, and End Results (SEER) program. Survival analysis was performed on the cases, and subgroups, using the Kaplan‐Meier and Cox regression methods. The concordance index of the Stage Size Grade Necrosis (SSIGN), Leibovich, and the UCLA integrated staging system, scores was determined to evaluate the accuracy of these outcome prediction models.

**Results:**

The 5‐year overall survival rate for RCC cases in West China Hospital was 87.6%; this was higher than that observed for SEER cases. Survival analysis identified several factors that exerted significant influence over prognosis, including the time of surgery, Eastern Cooperative Oncology Group performance status, tumor stage, size, nuclear differentiation, pathological subtypes, along with necrotic and sarcomatoid differentiation. Moreover tumor stage, size, and nuclear grade were all identified as independent predictors for both our cases and those from the SEER program. Patient groups with advanced RCC, and poorly differentiated RCC subgroups, were both determined to have a poor prognosis. The SSIGN model yielded the best predictive value as a prognostic model, followed by the Leibovich, and UCLA integrated staging system; this was the case for our patients, and for sub‐groups with a poor prognosis.

**Conclusion:**

The prognosis of RCC was mostly influenced by tumor stage, size, and nuclear differentiation. SSIGN may represent the most suitable prognostic model for the Chinese population.

## Graphical abstract

In our study, we aimed to investigate the survival characteristics of postoperative non‐metastatic renal cell carcinoma patients, and the predictive value of prognostic models. We retrospectively reviewed 1 202 postoperative RCC cases at the West China Hospital, Sichuan University (Chengdu, China), with 53 205 cases from the SEER database also reviewed as reference. Prognostic factors of renal cell carcinoma were analyzed in detail, and the prediction accuracy of the prognostic models was validated.

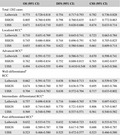

## 1 INTRODUCTION

Renal cell carcinoma (RCC) accounts for 2%‐3% of all malignant tumors in adults. Of all patients with RCC, approximately 30% have advanced‐stage RCC at the time of diagnosis. In addition, cancer metastasis usually occurs in 20%‐40% of patients after surgery, even in those with localized RCC.^1^ Considering the poor prognosis for patients with advanced RCC, surgery is insufficient and systemic adjuvant therapy may be useful to prolong survival.

As the standard treatment of advanced RCC, sunitinib is known to significantly prolong disease‐free survival (DFS), and overall survival (OS), compared with α‐ interferon.^2^ Furthermore, immune checkpoint inhibitors have shown superior treatment effects than standard target drugs in recent trails. For example, the two anti‐programmed death 1 (PD‐1) monoclonal antibodies, nivolumab and pembrolizumab, both demonstrated better survival rates than sunitinb, when combined with ipilimumab or axitinib separately,^3^, ^4^ and also better effects for the programmed death ligand 1 (PD‐L1) monoclonal antibody, avelumab, when combined with axitinib.^5^ However, as yet, trials have yet to demonstrate the benefit of adjuvant therapy in terms of survival, irrespective of whether we consider sorafenib, sunitinib, pazopanib, or axitinib.^6^, ^7^, ^8^ First, it is worth considering the initiation of trails to investigate therapeutic protocols for RCC that utilize adjuvant immune checkpoint inhibitors. Second, it is reasonable to hypothesize that more rational stratification of adjuvant therapies might lead to specific patients receiving greater levels of benefit.

Several studies have examined and recommended prediction models for the outcome of patients with RCC that could classify patients with differing probabilities of prognosis.^9^, ^10^, ^11^ Prediction models might have the potential to determine the need for systemic therapy, develop postoperative surveillance programs, and stratify patients for clinical trials. These models used a range of clinical and pathological variables, which were weighted in different ways, to predict outcomes.

The UCLA integrated staging system (UISS), which was developed by Zisman et al, combines the Tumor, Node, Metastasis (TNM) staging system, Fuhrman grade, and Eastern Cooperative Oncology Group (ECOG) performance status, to classify patients into five prognostic groups.^11^ Frank et al developed the Mayo Clinic Stage Size Grade Necrosis (SSIGN) score, which also incorporates the TNM stage, tumor size, nuclear grade, tumor necrotic and metastatic status, and stratifies patients into three risk groups.^10^ The Leibovich score, a modified variant of the SSIGN score, considers all of the variants included in the SSIGN, except for metastatic status. Internal validations of these three prediction models demonstrated their feasibility, while external validations were performed by several researchers.^9^ However, these validations were mostly based on the Caucasian population. In the present study, we performed survival analysis for 1202 cases of postoperative clinical nonmetastatic RCC. To allow comparison, we also acquired data from a large number of cases that featured in the National Cancer Institute's Surveillance, Epidemiology, and End Results (SEER) Program. We then carried out external validation of the scores derived from the UISS, SSIGN, and Leibovich, model on both own patients and subgroups from the SEER program. We sought to identify factors influencing the prognosis of postoperative Chinese patients with RCC, and assess the prognostic value of a range of predictive models for this subset of patients.

## 2 MATERIALS AND METHODS

### 2.1 Patient selection

We selected clinically nonmetastatic patients who underwent radical nephrectomy, or partial nephrectomy, for unilateral RCC between 1999 and 2012 in the Department of Urology of West China Hospital (WCH), Sichuan University (Chengdu, China). We excluded patients with bilateral renal neoplasms, an ECOG score >1, and those not willing to provide information regarding their disease. Patients provided informed written consent prior to the collection of data. Our final analysis included 1,202 patients with clinically nonmetastatic RCC.

### 2.2 Clinical and pathological features

Patient clinical data (eg, gender, age, preoperative ECOG score, smoking history, and diseases of the heart and cerebral vessels) were retrieved from the medical records of our hospital. Pathological reports were all provided by the Pathology Department of our institute, including histopathological type, pathological T stage and lymph status, sarcomatoid and cystic differentiation status, tumor size, nuclear grade, and necrotic status. Pathological T and lymph stage was adjusted according to the 8th American Joint Committee on Cancer TNM classification system,^12^ and the largest diameter of the tumor was recorded to determine its size. In accordance with TNM staging, RCC patients were divided into localized RCC (T_1‐2_N_0_M_0_) and advanced RCC (T_3‐4_N_0_M_0_ or T_any_N_+_M_x_). The Fuhrman nuclear grade 13 was reported according to the 2012 International Society of Urological Pathology consensus.^14^ Tumor necrosis, which was defined as microscopic coagulative necrosis, was reported during pathological diagnosis. Pathology reports also featured information relating to cystic architecture and the sarcomatoid component.

### 2.3 Follow‐up of patients

Chest and abdominal computerized tomography (CT) was performed 3 months after surgery. Subsequently, abdominal ultrasound or CT was performed twice annually for the first 5 years, and once annually thereafter. Chest X‐ray examinations were performed annually. Furthermore, all patients received a follow‐up telephone call each year.

### 2.4 The SEER data resource and cohort selection

We collected data from patients diagnosed with RCC between 2010 and 2015 and recorded on the National Cancer Institute's SEER program. We extracted data from all cases initially diagnosed with RCC, and selected nonmetastatic cases who underwent surgery for RCC. A total of 53 205 cases were included in our final analysis; all of these cases had a complete set of data available for evaluation, including age at diagnosis, gender, race, tumor TNM stage, nuclear grade, histological types, and survival status.

### 2.5 Statistical methods

For WCH patients, we used the Kaplan‐Meier method to estimate OS, DFS, and cancer‐specific survival (CSS). The duration of the follow‐up period was from the day of surgery to the day of patient death, or the last clinical evaluation. The endpoint of DFS was defined as the detection of recurrence or metastasis during the follow‐up period. CSS was defined as patient death caused by the recurrence of RCC, or metastasis, or any relevant complication. Differences in survival between two or more subgroups were evaluated using log‐rank tests. Univariate and multivariate Cox regression analyses were performed to determine the clinicopathological parameters associated with the survival of RCC patients. Patients were stratified using the SSIGN, Leibovich, and UISS scores,^9^, ^10^, ^11^ and the predictive ability of these outcome prediction models was evaluated using the concordance index (c‐index).^15^

Case information retrieved from the SEER program was analyzed using the Kaplan‐Meier method, and differences between the subgroups were evaluated via log‐rank tests. Univariate and multivariate Cox regression analyses were also used to determine the clinicopathological parameters associated with the survival of RCC patients.

Statistical analyses were performed using the IBM SPSS Statistics 2 (IBM SPSS Statistics) and The R Programming Language 3.5.0 (R Foundation for Statistical Computing). Differences were considered to be statistically significant when *P* < .05.

## 3 RESULTS

The clinicopathological data of 1,202 cases of postoperative clinically nonmetastatic RCC are shown in Table 1. The mean age was 55.5 years (standard deviation: 13.04 years), and the median follow‐up time was 63.02 months (interquartile range: 47.2‐83.4 months). Survival analysis showed that the 2‐ and 5‐year survival rates for OS, DFS, and CSS were 94.7% and 87.6%, 92.8% and 82.5%, and 94.9% and 88.1%, respectively. The clinicopathological parameters for the 53,205 cases selected from the SEER database are shown in Table 2. The median follow‐up time for these cases was 28.0 months (interquartile range: 12.0‐47.0 months), while the 2‐ and 5‐year OS and CSS were 91.1% and 80.0%, and 96.1% and 92.3%, respectively. The proportions of cases with advanced RCC, and poor differentiated RCC, were both higher for the SEER cases than the WCH cases.

**Table 1:** Clinical and pathological features of 1202 patients who underwent radical or partial nephrectomy for unilateral RCC in WCH

Clinical variants	n	Percentage (%)	5‐y survival rate^a^
Gender			
Male	750	62.4	86.3
Female	452	37.6	89.8
Preoperative age (y)			
≤50	447	37.2	91.1
>50	755	62.8	85.4
ECOG			
0	821	68.3	89.4
1	381	31.7	83.5
Smoking history			
No	893	74.3	87.8
Yes	309	25.7	86.8
Heart and cerebral vessels disease			
No	972	80.9	87.5
Yes	230	19.1	87.9
Histopathological type			
ccRCC	1 066	88.7	88.1
Papillary RCC	43	3.6	75.6
Chromophobe RCC	51	4.2	96.1
Other type	42	3.5	75.2
Postoperative T stage			
pT_1_	921	76.6	92.6
pT_2_	149	12.4	75.1
pT_3_	89	7.4	70.7
pT_4_	43	3.6	57.6
Postoperative N stage			
pN_0_ + pN_x_	1146	95.3	89.6
pN_1_	56	4.7	44.3
Metastasis during follow‐up			
M0 + Mx	1101	91.6	91.9
M1	101	8.4	34.3
Tumor size			
<5 cm	629	52.3	95.0
5‐10 cm	473	39.4	82.4
≥10 cm	88	7.3	63.4
Unrecorded	12	1.0	75.0
Advanced RCC			
No	1030	85.7	91.8
Yes	172	14.3	61.8
Fuhrman nuclear grade			
Grade 1	43	3.6	100.0
Grade 2	544	45.3	94.1
Grade 3	530	44.1	84.7
Grade 4	85	7.1	56.1
Necrosis			
No	1008	83.9	90.5
Yes	194	16.1	72.2
Sarcomatoid differentiation			
No	1180	98.2	88.0
Yes	22	1.8	61.0
Cystic differentiation			
No	1153	95.9	87.1
Yes	49	4.1	98.0

Note

n: number of patients.

a Percentage of patient survival 5 y after surgery;

**Table 2:** Clinical and pathological feature of 53 205 postoperative RCC patients whose datum were recorded in SEER database

Clinical and pathological variants	n	Percentage (%)	5‐y survival rate^a^
Gender			
Male	33 612	63.2	79.0%
Female	19 593	36.8	81.8%
Preoperative age (y)			
≤50	10 074	18.9	91.6
>50	43 131	81.1	77.4
Race			
White	43 172	81.1	79.6
Black	6252	11.8	81.2
Asian and Pacific islander	2880	5.4	81.8
American Indians and Alaska native	494	0.9	82.5
Unknown	407	0.8	97.4
Histopathological type			
ccRCC	33 078	62.2	82.5
Papillary RCC	6912	13.0	82.1
Chromophobe RCC	2322	4.4	89.7
Other type	10 893	20.5	69.7
T stage			
T_1_	37 186	69.9	85.9
T_2_	5310	10.0	79.1
T_3_	10 050	18.9	62.9
T_4_	659	1.2	20.2
N stage			
N_0_	51 989	97.7	81.2
N_+_	1216	2.3	31.8
Tumor size			
<5 cm	32 851	61.7	85.2
5‐10 cm	16 071	30.2	74.6
≥10 cm	4013	7.5	62.8
Unknown	270	0.5	49.1
Advanced RCC			
No	42 212	79.3	85.2
Yes	10 993	20.7	60.6
Nuclear grade			
Grade 1	6134	11.5	86.5
Grade 2	27 292	51.3	85.6
Grade 3	15 036	28.3	76.2
Grade 4	4743	8.9	50.0

Note

n: number of patients.

a Percentage of patient survival 5 y after surgery

For the 1,202 postoperative clinically nonmetastatic RCC patients, our univariate analysis (Table 3) revealed that older age, higher ECOG, higher TNM stage and Fuhrman grade, larger size, papillary RCC, and the presence of necrotic and sarcomatoid differentiation, were significantly correlated with poor survival (*P* < .05). In contrast, gender, smoking history, medical complications, and cystic differentiation, were demonstrated to be factors that were not significantly associated with survival (*P* > .05). Multivariate analysis (Table 4) showed that tumor stage (T_3_, T_4_, N_+_), tumor nuclear grade, and tumor size, were independent predictors of survival.

**Table 3:** Univariate analysis of 1202 cases of postoperative nonmetastatic RCC patients in WCH

Clinical pathological data	OS		DFS		CSS	
	*P* value	Hazard ratio (95% CI)	*P* value	Hazard ratio (95% CI)	*P* value	Hazard ratio (95% CI)
Male^a^	.172	1.260 (0.904‐1.757)	.479	1.103 (0.841‐1.445)	.245	1.221(0.872‐1.710)
Preoperative age >50 y^b^	.001	1.788 (1.249‐2.559)	.002	1.550 (1.161‐2.069)	.003	1.752 (1.217‐2.521)
ECOG^c^	.005	1.578 (1.148‐2.169)	.003	1.488 (1.140‐1.943)	.013	1.511 (1.091‐2.093)
Smoking history^d^	.830	1.040 (0.728‐1.485)	.830	1.048 (0.773‐1.420)	.792	1.051 (0.725‐1.523)
Heart and cerebral vessels disease^e^	.679	1.085 (0.736‐1.600)	.774	1.051 (0.749‐1.474)	.643	1.098 (0.740‐1.630)
pT_2_ f	.000	3.344 (2.253‐4.962)	.000	3.211 (2.315‐4.453)	.000	3.596 (2.412‐5.362)
pT_3_ f	.000	4.650 (3.010‐7.185)	.000	4.303 (2.977‐6.218)	.000	4.991 (3.216‐7.745)
pT_4_ f	.000	7.653 (4.620‐12.677)	.000	6.942 (4.496‐10.719)	.000	7.568 (4.502‐12.722)
pN+^f^	.000	6.589 (4.399‐9.869)	.000	6.205 (4.496‐10.719)	.000	6.880 (4.585‐10.325)
Tumor size 5‐10 cm^g^	.000	3.626 (2.451‐5.365)	.000	2.742 (2.016‐3.729)	.000	3.708 (2.481‐5.544)
Tumor size ≥10 cm^g^	.000	7.925 (4.905‐12.803)	.000	6.388 (4.328‐9.428)	.000	8.407 (5.168‐13.676)
Nuclear intermediate differentiation^h^	.000	2.591 (1.767‐3.798)	.000	2.666 (1.952‐3.640)	.000	2.824 (1.895‐4.210)
Nuclear poor differentiation^h^	.000	8.339 (5.261‐13.216)	.000	6.536 (4.369‐9.777)	.000	9.313 (5.802‐14.951)
Papillary RCC^i^	.050	1.900 (1.000‐3.613)	.007	2.057 (1.216‐3.479)	.036	1.988 (1.045‐3.783)
Chromosome RCC^i^	.095	0.305 (0.075‐1.231)	.164	0.532 (0.219‐1.293)	.105	0.315 (0.078‐1.273)
Other type^i^	.018	2.178 (1.145‐4.141)	.001	2.362 (1.396‐3.996)	.012	2.273 (1.194‐4.326)
Sarcomatoid differentiation^j^	.000	3.682 (1.807‐7.500)	.002	2.910 (1.493‐5.668)	.000	3.837 (1.882‐7.823)
Necrosis^k^	.000	2.724 (1.947‐3.812)	.000	2.643 (1.991‐3.509)	.000	2.890 (2.059‐4.058)
Cystic differentiation^l^	.052	0.142 (0.020‐1.017)	.063	0.392 (0.146‐1.053)	.057	0.148 (0.021‐1.059)

Abbreviations: CSS: cancer‐specific survival; DFS: disease‐free survival; OS: overall survival.

a Reference group is female.

b Reference group is preoperative age ≤50.

c Reference group is ECOG = 0.

d Reference group is no smoker.

e Reference group is no complication.

f Reference group for T stage is pT_1_ and N stage is N_0_ or N_x_.

g Reference group is tumor size <5 cm.

h Reference group is nuclear well differentiation RCC.

i Reference group is ccRCC.

j Reference group is no sarcomatoid differentiation.

k Reference group is no necrosis.

l Reference group is no cystic differentiation.

**Table 4:** Multivariate analysis of 1202 cases of WCH

Clinical pathological data^a^	OS		DFS		CSS	
	*P* value	Hazard ratio (95% CI)	*P* value	Hazard ratio (95% CI)	*P* value	Hazard ratio (95% CI)
Preoperative age >50	.261	1.250 (0.847‐1.846)	.539	1.106 (0.802‐1.523)	.304	1.230 (0.829‐1.824)
ECOG	.587	1.100 (0.780‐1.551)	.342	1.153 (0.860‐1.546)	.736	1.062 (0.748‐1.507)
pT_2_	.208	1.373 (0.838‐2.251)	.069	1.470 (0.970‐2.228)	.153	1.440 (0.873‐2.374)
pT_3_	.001	2.367 (1.448‐3.868)	.000	2.426 (1.595‐3.689)	.000	2.473 (1.506‐4.063)
pT_4_	.000	4.315 (2.467‐7.548)	.000	3.789 (2.330‐6.160)	.000	4.101 (2.309‐7.283)
pN+	.000	2.975 (1.889‐4.683)	.000	2.756 (1.839‐4.131)	.000	2.971 (1.880‐4.696)
Tumor size 5‐10 cm	.000	2.403 (1.580‐3.653)	.000	1.880 (1.349‐2.621)	.000	2.409 (1.566‐3.705)
Tumor size ≥10cm	.000	3.192 (1.700‐5.993)	.000	2.533 (1.504‐4.266)	.000	3.181 (1.679‐6.027)
Nuclear intermediate differentiation	.006	1.773 (1.176‐2.671)	.000	1.929 (1.382‐2.692)	.004	1.870 (1.223‐2.858)
Nuclear poor differentiation	.000	3.206 (1.845‐5.570)	.000	2.524 (1.557‐4.090)	.000	3.450 (1.965‐6.057)
Papillary RCC	.770	1.106 (0.562‐2.175)	.299	1.341 (0.771‐2.332)	.641	1.175 (0.597‐2.311)
Chromosome RCC	.156	0.359 (0.087‐1.476)	.197	0.551 (0.223‐1.363)	.137	0.343 (0.083‐1.407)
Other type	.519	1.273 (0.612‐2.648)	.104	1.633 (0.904‐2.948)	.506	1.284 (0.614‐2.686)
Necrosis	.226	1.268 (0.864‐1.862)	.087	1.325 (0.959‐1.830)	.209	1.282 (0.870‐1.889)
Sacromatoid differentiation	.681	0.838 (0.362‐1.940)	.589	0.810 (0.377‐1.742)	.670	0.833 (0.359‐1.932)

a Reference groups are the same as described inTable 3.

For the 53,205 SEER cases, univariate analysis showed that gender, age, TNM stage, nuclear grade, size, and pathological subtypes, were all prognostic factors of RCC (*P* < .05, Table 5). Furthermore, these factors were also independent predictors of outcome (Table 6).

**Table 5:** Univariate analysis of 53,205 cases of postoperative nonmetastatic RCC patients from the SEER database

Clinical pathological data	OS		CSS	
	*P* value	Hazard ratio (95% CI)	*P* value	Hazard ratio (95% CI)
Male^a^	.000	1.173 (1.112‐1.237)	.000	1.155 (1.061‐1.258)
Preoperative age >50 years^b^	.000	3.059 (2.778‐3.367)	.000	2.169 (1.898‐2.479)
Black^c^	.106	0.937 (0.865‐1.014)	.001	0.799 (0.698‐0.915)
Asian and Pacific Islander^c^	.325	0.943 (0.839‐1.06)	.415	1.075 (0.904‐1.279)
American Indians and Alaska native^c^	.993	0.999 (0.769‐1.297)	.073	0.618 (0.365‐1.045)
pT_2_ d	.000	1.723 (1.583‐1.875)	.000	3.838 (3.337‐4.403)
pT_3_ d	.000	3.508 (3.318‐3.708)	.000	9.181 (8.318‐1‐0.135)
pT_4_ d	.000	15.357 (13.818‐17.069)	.000	45.295 (39.032‐52.564)
pN+^d^	.000	7.210 (6.624‐7.848)	.000	13.450 (12.088‐14.966)
Tumor size 5‐10 cm^e^	.000	1.940 (1.836‐2.051)	.000	4.152 (3.752‐4.594)
Tumor size ≥10 cm^e^	.000	3.435 (3.192‐3.698)	.000	11.027 (9.863‐12.329)
Nuclear intermediate differentiation^f^	.000	1.982 (1.870‐2.100)	.000	4.012 (3.613‐4.455)
Nuclear poor differentiation^f^	.000	5.703 (5.350‐6.080)	.000	14.546 (13.069‐16.190)
Papillary RCC^g^	.032	1.093 (1.008‐1.186)	.016	0.836 (0.723‐0.967)
Chromosome RCC^g^	.000	0.575 (0.484‐0.684)	.000	0.373 (0.265‐0.524)
Other type^g^	.000	2.224 (2.105‐2.349)	.000	2.427 (2.228‐2.643)

Abbreviations: OS, overall survival; CSS, cancer‐specific survival.

a Reference group is female.

b Reference group is preoperative age ≤50 y.

c Reference group is White RCC patients.

d Reference group for T stage is pT_1_ and N stage is N_0_ or N_x_.

e Reference group is tumor size <5 cm.

f Reference group is nuclear well differentiation RCC.

g Reference group is ccRCC.

**Table 6:** Multivariate analysis of 53 205 cases of SEER

Clinical pathological data	OS		CSS	
	*P* value	Hazard ratio (95% CI)	*P* value	Hazard ratio (95% CI)
Male	.001	0.912 (0.864‐0.963)	.864	0.992 (0.910‐1.082)
Preoperative age >50	.000	2.469 (2.241‐2.720)	.000	1.579 (1.380‐1.808)
Black	.160	1.061 (0.977‐1.152)	.998	1.000 (0.869‐1.150)
Asian and Pacific Islander	.452	0.956 (0.850‐1.075)	.443	1.071 (0.900‐1.274)
American Indians and Alaska native	.460	1.105 (0.849‐1.438)	.104	0.636 (0.369‐1.098)
pT_2_	.052	1.101 (0.999‐1.214)	.000	1.520 (1.300‐1.778)
pT_3_	.000	1.891 (1.764‐2.027)	.000	3.037 (2.690‐3.429)
pT_4_	.000	4.675 (4.121‐5.303)	.000	7.296 (6.089‐8.742)
pN+	.000	2.276 (2.070‐2.504)	.000	2.725 (2.419‐3.069)
Tumor size 5‐10 cm	.000	1.342 (1.260‐1.428)	.000	2.135 (1.908‐2.390)
Tumor size ≥10 cm	.000	1.790 (1.636‐1.958)	.000	3.588 (3.136‐4.106)
Nuclear intermediate differentiation	.000	1.432 (1.347‐1.523)	.000	2.240 (2.007‐2.500)
Nuclear poor differentiation	.000	2.455 (2.272‐2.654)	.000	4.216 (3.723‐4.775)
Papillary RCC	.000	1.167 (1.073‐1.271)	.490	1.055 (0.907‐1.227)
Chromosome RCC	.000	0.577 (0.485‐0.685)	.000	0.353 (0.251‐0.496)
Other type	.000	1.461 (1.375‐1.553)	.000	1.346 (1.223‐1.482)

Note

Reference groups are the same as described in Table 5.

Both TNM stage and nuclear grade are included in the SSIGN, Leibovich, and UISS outcome prediction models, which were used in this study to classify patients according to different survival outcomes. The c‐index of each model indicated that for the prediction of OS, DFS, and CSS, in postoperative clinically nonmetastatic RCC patients, the SSIGN score offered the highest discrimination among the three models. Notably, the Leibovich score was slightly inferior to the SSIGN score; for our cases, we also found that discrimination of the UISS was poor (Table 7).

**Table 7:** Predictive ability of different models on 1202 RCC cases

	OS (95% CI)		DFS (95% CI)		CSS (95% CI)	
Total 1202 cases						
Leibovich	0.773	0.728‐0.818	0.754	0.717‐0.793	0.782	0.736‐0.828
SSIGN	0.805	0.760‐0.850	0.798	0.760‐0.835	0.817	0.772‐0.863
UISS	0.671	0.632‐0.710	0.653	0.620‐0.686	0.674	0.633‐0.714
Localized RCC^a^						
Leibovich	0.710	0.651‐0.769	0.693	0.645‐0.741	0.723	0.663‐0.784
SSIGN	0.745	0.686‐0.804	0.744	0.696‐0.791	0.765	0.705‐0.825
UISS	0.653	0.601‐0.704	0.622	0.580‐0.664	0.661	0.609‐0.714
Advanced RCC^a^						
Leibovich	0.662	0.591‐0.733	0.649	0.586‐0.711	0.670	0.598‐0.741
SSIGN	0.762	0.690‐0.834	0.752	0.688‐0.815	0.765	0.692‐0.837
UISS	0.494	0.434‐0.555	0.494	0.4410‐0.548	0.505	0.443‐0.566
Well‐differentiated^a^ RCC						
Leibovich	0.662	0.591‐0.733	0.638	0.564‐0.713	0.634	0.539‐0.729
SSIGN	0.674	0.588‐0.760	0.707	0.636‐0.778	0.695	0.603‐0.786
UISS	0.704	0.624‐0.783	0.638	0.572‐0.704	0.717	0.633‐0.802
Intermediate‐ differentiated RCC^a^						
Leibovich	0.757	0.696‐0.818	0.716	0.666‐0.765	0.759	0.697‐0.821
SSIGN	0.805	0.744‐0.865	0.770	0.721‐0.819	0.806	0.745‐0.867
UISS	0.569	0.548‐0.590	0.554	0.537‐0.571	0.561	0.540‐0.582
Poor‐differentiated RCC^a^						
Leibovich	0.632	0.533‐0.731	0.632	0.540‐0.723	0.632	0.533‐0.731
SSIGN	0.688	0.589‐0.787	0.708	0.617‐0.799	0.688	0.589‐0.787
UISS	0.523	0.466‐0.580	0.525	0.472‐0.577	0.523	0.466‐0.580

a Subgroup of total 1202 cases; CI: confidence interval; Leibovich: Leibovich RCC score; SSIGN: stage, size, grade, and necrosis; UISS, University of California Los Angeles Integrated Staging System.

In our study, we observed poor survival rates in subgroups of patients with advanced RCC, and poorly differentiated nuclear grade (Fuhrman IV); there was no significant difference in terms of survival (*P* < .05) between these two groups for both WCH and SEER cases. Multivariate analysis was performed in these two subgroups; we found that tumor N stage, and size, were independent predictors for each subgroup of WCH cases, while age, tumor stage, size, nuclear differentiation, and pathological subtypes were independent predictors for the SEER cases (Supplemental Material Table S1).

The SSIGN, Leibovich, and UISS, scores were used to stratify RCC patients with different tumor stages and tumor nuclear grades. The c‐index for each prediction model across different subgroups is shown in Table 7. Results suggested that the predictive effects observed in the subgroups were weaker compared to those reported for the total cohort of cases. The SSIGN and Leibovich scores performed well for localized RCC, while only SSIGN showed acceptable discrimination for advanced RCC. However, when analyzing different nuclear grade subgroups, the discrimination offered by SSIGN and Leibovich for the group of patients with intermediate differentiation was higher than that recorded for the well‐differentiated group. The UISS score exhibited an inverse effect. The predictive effect of all three models in poorly differentiated RCC was weak, with a c‐index for SSIGN approximating 0.70.

## 4 DISCUSSION

RCC ranks second among urinary neoplasms (after bladder cancer), with a 5‐year survival rate of only 71%.^16^ The prognosis of RCC is influenced by numerous factors, including age at the time of operation, preoperative performance status, laboratory examination results, pathological tumor stage, nuclear grade, and tumor histological subtype^17^; these parameters can be classified into clinical and pathological prognostic factors. In the present study, age at the time of operation, ECOG, tumor stage, size, nuclear differentiation, pathological subtype, and necrotic and sarcomatoid differentiation, were identified as prognostic factors for clinically nonmetastatic RCC; other factors showed no definitive association.

Gender is a key factor of interest. In a previous research study, Kutikov et al demonstrated poorer survival for males in cases that were registered with SEER in 2010.^18^ However, studies by Xu et al, and Zhu et al, involving 378 and 1108 Chinese patients, respectively, failed to find any association with gender.^19^, ^20^ These previous findings concurred with our present findings, which failed to identify any significant association for gender in either the WCH or SEER cases (2018). Therefore, it can be hypothesized that the influence of gender on patient survival exhibits differences between Chinese and Caucasian cases.

In terms of racial influences, Stafford et al retrospectively analyzed 39,434 American patients with RCC. Interestingly, higher incidence rates, and lower survival rates, were reported in Black patients, while Asian patients were associated with a better prognosis.^21^ Similar results were also demonstrated by Rose et al, who analyzed 48,846 cases from the National Cancer Data Base. Although there was no significant difference, this study reported contradictory results to those observed in our current research involving 53 205 SEER cases. We observed that the prognosis of Caucasians was poor, while similar levels of survival were apparent for Black and Asian patients. Further studies are now needed to investigate the specific impact of race on prognosis.

Postoperative pathological examination results (for example, tumor stage, size, nuclear differentiation, pathological subtype, and special differentiation) are generally accepted as being the key determinants for the prognosis of patients with RCC. In line with previous findings,^22^ our current research identified that pathological stage was the single most crucial prognostic factor for RCC; a poor RCC stage is negatively correlated with survival. Invasion of the perinephric fat, collecting system, and the adrenal and venous system, have been associated with poorer prognosis in otherwise organ‐confined RCC.^17^, ^23^, ^24^, ^25^, ^26^, ^27^, ^28^ In addition, the nuclear grade plays a significant role in the prognostic prediction of RCC.^17^, ^29^ The Fuhrman nuclear grade is the most widely adopted grading system for RCC. In 1997, the World Health Organization classified Fuhrman I and II as “well differentiated,” Fuhrman III as “intermediately differentiated,” and Fuhrman IV as “poorly differentiated.” Significant prognostic differences were observed in the present when compared with many other studies.^9^, ^10^ A number of urological guidelines have recommended using the World Health Organization system.^30^ Although there were overlaps of concept with regard to tumor size and stage, we identified that tumor size was an independent predictor. The independent predictive value of tumor stage, size, and nuclear differentiation, was demonstrated in both WCH and SEER cases, as well as in many previous studies.^9^, ^10^ It is therefore advisable to incorporate these factors into models when predicting the prognosis of RCC.

The UISS scoring system, which incorporates tumor stage, Fuhrman nuclear grade, and ECOG, was designed to predict the OS of RCC patients with or without metastasis.^11^ This system stratifies patients into six prognostic groups, with significant prognostic differences between each group.^11^ When initially proposed, this system was intended to be an accurate model. However, discrepant results were obtained from different external validation studies, with the discrimination ranging between 0.58 and 0.86.^29^, ^31^, ^32^, ^33^, ^34^ In the study conducted by Lv et al, the discrimination offered by the UISS model was 0.64 (95% confidence interval: 0.586‐0.693),^34^ which was similar to that reported in our current research. This suggests that the UISS model may not be suitable for use in Chinese postoperative patients with RCC.

The SSIGN score incorporates tumor TNM stage, nuclear grade, tumor size, and pathological necrosis. This stratifies patients into three risk groups, and finally predicts RCC metastasis and survival.^10^ Zigeuner et al performed an external validation showing a c‐index of 0.823 in 1,862 European cases. Considerable discrimination was also demonstrated in 1,795 American contemporary patients.^35^, ^36^ In 2008, Fujii et al also reported an acceptable c‐index of 0.814 in 401 Japanese cases.^37^ In our external validation, SSIGN did not offer a high degree of discrimination (c‐index >0.9) for OS, DFS, or CSS. However, all of our c‐indices were >0.75, and were therefore similar to the results reported by Fujii et al.^37^ We therefore consider that the SSIGN score may represent a suitable prediction model for Caucasians, as well as East Asian patients.

The Leibovich‐revised SSIGN score was proposed in 2003; in this model, the metastatic tumor status was eliminated, the cut‐off point for tumor size was modulated, and the weighting for T_2‐4_ was increased.^9^ In this model, the metastatic risk of postoperative RCC patients, rather than their survival, was the primary predictive goal. Internal validation yielded a discrimination of 0.819, while external validations, performed in different clinical centers, demonstrated a c‐index between 0.740 and 0.864^38^, ^39^, ^40^, ^41^; these values seem acceptable. Tan et al applied the Leibovich score to predict OS and DFS in Singaporean RCC patients and found that the prediction effect was poor (c‐index for OS and DFS: 0.670 and 0.700, respectively).^40^, ^41^ However, in our postoperative nonmetastatic RCC patients, the c‐index of the Leibovich score was lower than the SSIGN score. Therefore, the application of the Leibovich score in Chinese individuals requires further investigation.

Although most of the prediction models performed well during internal validation, the results arising from external validation were controversial. We hypothesized that the application of prognostic models was limited by the relatively small sample size for modeling populations, and by heterogeneity between the modeling population and prediction population. First, most of the current prognostic models were established based on approximately 1000 cases, for whom prognostic factors were recorded in detail. Although data samples from large databases, such as the SEER, are sufficient, some of the prognostic factors are often insufficiently recorded. Second, there was clear heterogeneity in our study when the WCH and SEER cases were compared. This heterogeneity between groups, especially the proportions of patients in the subgroup with a poor prognosis; this may had led to differences in survival. Therefore, it is rational to speculate that inter‐population heterogeneity may influence the predictive accuracy of these prognostic models.

Both advanced RCC, and poor nuclear differentiated RCC, were linked to a poor prognosis. There was no significant difference in terms of survival between these two subgroups, neither in the WCH cases nor in the SEER cases, which shared similar curves for survivorship. In a previous study, Wloff et al defined the high‐risk group as patients with a tumor of either stage pT3 with a diameter >7 cm, stage ≥pT3b, pN+ stage, or Fuhrman grade III‐IV. This group was demonstrated better than differentiation methods based on inclusion criteria of current clinical trials on adjuvant treatment.^42^ Based on these risk criteria, Bandini et al found that poor prognosis was associated with number of risk factors.^43^ Although different demarcation points were selected, results showed that tumor stage, nuclear differentiation grade, and tumor size, could be used to distinguish between patients with poor and good prognosis, and subsequently allow the individualization of adjuvant therapy.

Prognosis models that are based on these variants are intended to classify subgroups of patients with a poor prognosis. The application of prediction models in subgroups of patients with RCC may result in the loss of variables, thus causing negative impact on the discriminatory power of these models. However, by applying more rational weighting on variables, it may be possible to create a more appropriate model for prediction. In this study, we applied the SSIGN, Leibovich, and UISS scores, on subgroups of patients with poor prognosis (for example, those with advanced and poorly differentiated RCC). Only the SSIGN score showed acceptable levels of discrimination. This suggests that SSIGN may be more suitable to discriminate patients with a poor prognosis.

At present, there is a strong rationale for systemic adjuvant therapy in patients with a poor prognosis. However, there are no clinical data at present to demonstrate the positive effect of adjuvant therapy on survival. However, we hypothesize that the administration of effective systemic therapy in patients with a poor prognosis, including advanced RCC and poorly differentiated RCC, is an important influential factor for prognosis. More rigorously designed clinical trials are now needed to test this hypothesis.

There are some limitations in our study that need to be considered. First, considering the principle underlying the calculations involved in the outcome prediction model, the gradient of variability guarantee the distinction of patients with different survival outcomes. Thus, there is an inherent limitation with respect to the prediction of survival in specific subgroups; the model predominantly shows poor discrimination. Second, population heterogeneity is inevitable in the comparison of WCH and SEER cases; such heterogeneity cannot be explained using the limited number of variables and sample size in the present study. Further research is required to fully investigate the effect of heterogeneity.

## 5 CONCLUSION

Age at the time of surgery, ECOG, tumor stage, size, nuclear differentiation, pathological subtypes, and both necrotic and sarcomatoid differentiation, were identified as exerting significant influence on the prognosis of postoperative patients with clinically nonmetastatic RCC. Moreover tumor stage, size, and nuclear differentiation, were identified as independent predictors of survival. Of the prediction models investigated herein, the SSIGN score may be more suitable for use in Chinese patients with RCC.

## Data Availability

The data that support the findings of this study are available on request from the corresponding author. The data are not publicly available due to privacy restrictions.
