# Supplementary material for: Prognostic analysis of postoperative clinically nonmetastatic renal cell carcinoma
Source: Cancer Med. 2019 Dec 16;9(3):959–70. doi: 10.1002/cam4.2775 (PMC6997064; doi:10.1002/cam4.2775)
Supplement: Supplementary file 1 [file CAM4-9-959-s001.docx]

**Table S1. Univariate analysis of advanced RCC of WCH**

| Clinical pathological datas* | OS | | DFS | | CSS | |
| --- | --- | --- | --- | --- | --- | --- |
|  | P value | Hazard ratio (95% CI) | P value | Hazard ratio (95% CI) | P value | Hazard ratio (95% CI) |
| Preoperative age > 50 | 0.689 | 0.899 (0.532 - 1.517) | 0.575 | 1.140 (0.721 - 1.803) | 0.812 | 1.067 (0.626 - 1.818) |
| ECOG | 0.120 | 0.660 (0.391 – 1.114) | 0.158 | 0.726 (0.465 - 1.133) | 0.089 | 0.630 (0.369 - 1.073) |
| pT_2_ | 0.000 | 3.344 (2.253 - 4.962) | 0.000 | 3.211 (2.315 - 4.453) | 0.000 | 3.596 (2.412 - 5.362) |
| pT_3_ | 0.000 | 4.65 (3.01 - 7.185) | 0.000 | 4.303 (2.977 - 6.218) | 0.000 | 4.991 (3.216 - 7.745) |
| pT_4_ | 0.000 | 7.653 (4.62 - 12.677) | 0.000 | 6.942 (4.496 - 10.719) | 0.000 | 7.568 (4.502 - 12.722) |
| pN+ | 0.000 | 6.589 (4.399 - 9.869) | 0.000 | 6.205 (4.357 - 8.836) | 0.000 | 6.88 (4.585 - 10.325) |
| Tumor size 5-10 cm | 0.006 | 2.781 (1.332 – 5.804) | 0.029 | 1.904 (1.068 - 3.394) | 0.008 | 2.707 (1.294 - 5.663) |
| Tumor size ≥10 cm | 0.000 | 4.627 (2.101 - 10.189) | 0.000 | 3.259 (1.730- 6.142) | 0.000 | 4.724 (2.144 -10.408) |
| Nuclear intermediate differentiation | 0.022 | 2.339 (1.132 - 4.831) | 0.017 | 2.057 (1.138 – 3.715) | 0.027 | 2.272 (1.098 - 4.702) |
| Nuclear poor differentiation | 0.006 | 3.033 (1.370 - 6.716) | 0.016 | 2.292 (1.171 - 4.487) | 0.006 | 3.048 (1.377 - 6.750) |
| Papillary RCC | 0.295 | 1.526 (0.693 - 3.361) | 0.249 | 1.504 (0.751 - 3.014) | 0.266 | 1.567 (0.711 - 3.455) |
| Chromosome RCC | 0.678 | 0.657 (0.091 - 4.757) | 0.443 | 0.461 (0.064 - 3.322) | 0.577 | 0.569 (0.079 - 4.120) |
| Other type | 0.021 | 2.549 (1.154 - 5.631) | 0.092 | 1.956 (0.897 - 4.263) | 0.018 | 2.613 (1.181 - 5.777) |
| Sacromatoid differentiation | 0.109 | 1.900 (0.867 - 4.163) | 0.131 | 1.753 (0.846 - 3.632) | 0.098 | 1.942 (0.886 - 4.257) |
| Necrosis | 0.003 | 2.116 (1.292 - 3.468) | 0.001 | 2.069 (1.362 - 3.226) | 0.002 | 2.207 (1.343 - 3.626) |

*reference groups are the same as described in Table 3 and 5

**Table S2. Univariate analysis of poor differentiation RCC of WCH**

| Clinical pathological datas* | OS | | DFS | | CSS | |
| --- | --- | --- | --- | --- | --- | --- |
| Preoperative age > 50 | 0.311 | 0.691 (0.339 - 1.412) | 0.192 | 0.645 (0.334 - 1.247) | 0.311 | 0.691 (0.339 - 1.412) |
| ECOG | 0.037 | 0.458 (0.220 – 0.955) | 0.055 | 0.525 (0.272 - 1.015) | 0.089 | 0.037 (0.220 – 0.955) |
| pT_2_ | 0.913 | 1.055 (0.409 - 2.721) | 0.643 | 1.22 (0.526 - 2.829) | 0.913 | 1.055 (0.409 - 2.721) |
| pT_3_ | 0.137 | 1.807 (0.829 - 3.94) | 0.122 | 1.794 (0.856 - 3.762) | 0.137 | 1.807 (0.829 - 3.94) |
| pT_4_ | 0.247 | 2.081 (0.602 - 7.199) | 0.352 | 1.792 (0.525 - 6.121) | 0.247 | 2.081 (0.602 - 7.199) |
| pN+ | 0.025 | 2.264 (1.107 - 4.633) | 0.027 | 2.152 (1.093 - 4.239) | 0.025 | 2.264 (1.107 - 4.633) |
| Tumor size 5-10 cm | 0.060 | 2.786 (0.957 – 8.113) | 0.024 | 3.369 (1.175 – 9.662) | 0.060 | 2.786 (0.957 – 8.113) |
| Tumor size ≥10 cm | 0.011 | 4.581 (1.408 – 14.901) | 0.013 | 4.472 (1.375 - 14.541) | 0.011 | 4.581 (1.408 – 14.901) |
| Papillary RCC | 0.788 | 0.76 (0.104 - 5.583) | 0.644 | 0.625 (0.086 - 4.568) | 0.788 | 0.76 (0.104 - 5.583) |
| Chromosome RCC | 0.982 | - | 0.980 | - | 0.982 | - |
| Other type | 0.837 | 0.883 (0.269 - 2.894) | 0.635 | 0.752 (0.231 - 2.442) | 0.837 | 0.883 (0.269 - 2.894) |
| Sacromatoid differentiation | 0.969 | 1.019 (0.396 - 2.631) | 0.760 | 1.145 (0.480 - 2.727) | 0.969 | 1.019 (0.395 – 2.631) |
| Necrosis | 0.155 | 1.625 (0.833 - 3.172) | 0.111 | 1.650 (0.891 - 3.057) | 0.155 | 1.625 (0.833 - 3.172) |

*reference groups are the same as described in Table 3 and 5**Table S3. Univariate analysis of advanced RCC of SEER database**

| Clinical pathological datas* | OS | | CSS | |
| --- | --- | --- | --- | --- |
|  | P value | Hazard ratio (95% CI) | P value | Hazard ratio (95% CI) |
| Preoperative age > 50 | 0.000 | 1.840 (1.589 - 2.131) | 0.023 | 1.209 (1.026 - 1.424) |
| Male | 0.171 | 0.946 (0.873 - 1.025) | 0.197 | 0.933 (0.839 - 1.037) |
| pT2 | 0.144 | 1.369 (0.898 - 2.085) | 0.041 | 1.742 (1.023 - 2.967) |
| pT3 | 0.059 | 0.743 (0.546 - 1.012) | 0.129 | 0.727 (0.481 - 1.097) |
| pT4 | 0.000 | 3.137 (2.274 - 4.328) | 0.000 | 3.53 (2.303 - 5.41) |
| pN+ | 0.000 | 3.016 (2.754 - 3.303) | 0.000 | 3.907 (3.489 - 4.374) |
| Tumor size 5-10 cm | 0.000 | 1.278 (1.164 - 1.404) | 0.000 | 1.827 (1.584 - 2.107) |
| Tumor size ≥10 cm | 0.000 | 1.877 (1.690 - 2.084) | 0.000 | 3.548 (3.059 - 4.115) |
| Nuclear intermediate differentiation | 0.000 | 2.085 (1.876 - 2.318) | 0.000 | 3.027 (2.584 - 3.546) |
| Nuclear poor differentiation | 0.000 | 4.075 (3.666 - 4.529) | 0.000 | 6.341 (5.420 - 7.420) |
| Papillary RCC | 0.001 | 1.291 (1.104 - 1.51) | 0.004 | 1.339 (1.097 - 1.634) |
| Chromosome RCC | 0.000 | 0.53 (0.387 - 0.726) | 0.001 | 0.466 (0.302 - 0.719) |
| Other type | 0.000 | 2.495 (2.308 - 2.697) | 0.000 | 2.185 (1.97 - 2.424) |

*reference groups are the same as described in Table 3 and 5

**Table S4. Univariate analysis of poor differentiation RCC of SEER database**

| Clinical pathological datas* | OS | | CSS | |
| --- | --- | --- | --- | --- |
|  | P value | Hazard ratio (95% CI) | P value | Hazard ratio (95% CI) |
| Preoperative age > 50 | 0.000 | 1.622 (1.333 - 1.973) | 0.448 | 1.088 (0.875 - 1.352) |
| male | 0.738 | 1.018 (0.915 – 1.133) | 0.318 | 1.074 (0.934 - 1.236) |
| pT_2_ | 0.011 | 1.308 (1.063 - 1.609) | 0.001 | 1.647 (1.219 - 2.224) |
| pT_3_ | 0.000 | 2.314 (2.011 - 2.662) | 0.000 | 3.363 (2.725 - 4.151) |
| pT_4_ | 0.000 | 7.080 (5.927 - 8.456) | 0.000 | 10.927 (8.524 - 14.007) |
| pN+ | 0.000 | 2.814 (2.487 - 3.184) | 0.000 | 3.346 (2.867 - 3.906) |
| Tumor size 5-10 cm | 0.000 | 1.568 (1.388 – 1.772) | 0.000 | 2.425 (2.023 - 2.907) |
| Tumor size ≥10 cm | 0.000 | 2.219 (1.928 – 2.555) | 0.000 | 4.303 (3.548 - 5.219) |
| Papillary RCC | 0.352 | 0.867 (0.641 - 1.171) | 0.013 | 0.584 (0.383 - 0.892) |
| Chromosome RCC | 0.000 | 0.43 (0.272 - 0.681) | 0.007 | 0.5 (0.303 - 0.827) |
| Other type | 0.000 | 1.511 (1.35 - 1.692) | 0.587 | 1.04 (0.904 - 1.196) |

*reference groups are the same as described in Table 3 and 5

**Table S5. Multivariate analysis of advanced and poor differentiation RCC of WCH**

|  | Clinical pathological datas* | OS | | DFS | | CSS | |
| --- | --- | --- | --- | --- | --- | --- | --- |
|  |  | P value | Hazard ratio (95% CI) | P value | Hazard ratio (95% CI) | P value | Hazard ratio (95% CI) |
| Advanced  RCC | pT_2_ | 0.815 | 0.887 (0.325 - 2.423) | 0.608 | 0.787 (0.315 - 1.966) | 0.767 | 0.858 (0.312 - 2.36) |
|  | pT_3_ | 0.509 | 1.392 (0.521 - 3.721) | 0.626 | 1.25 (0.509 - 3.065) | 0.497 | 1.407 (0.525 - 3.771) |
|  | pT_4_ | 0.064 | 2.527 (0.947 - 6.743) | 0.097 | 2.104 (0.874 - 5.064) | 0.089 | 2.342 (0.877 - 6.252) |
|  | pN+ | 0.029 | 2.342 (1.091 - 5.027) | 0.028 | 2.233 (1.089 - 4.579) | 0.025 | 2.41 (1.116 - 5.201) |
|  | 5 cm≤tumor size＜10 cm | 0.022 | 2.396 (1.133 - 5.068) | 0.105 | 1.631 (0.903 - 2.945) | 0.029 | 2.31 (1.088 - 4.904) |
|  | Tumor size ≥10 cm | 0.004 | 3.615 (1.505 - 8.682) | 0.008 | 2.625 (1.291 - 5.338) | 0.004 | 3.681 (1.528 - 8.867) |
|  | Nuclear intermediate differentiation | 0.230 | 1.617 (0.738 - 3.542) | 0.172 | 1.599 (0.815 - 3.138) | 0.279 | 1.544 (0.703 - 3.39) |
|  | Nuclear poor differentiation | 0.115 | 2.048 (0.839 - 4.996) | 0.252 | 1.59 (0.719 - 3.516) | 0.128 | 2 (0.819 - 4.886) |
|  | Necrosis | 0.187 | 1.465 (0.831 - 2.585) | 0.114 | 1.503 (0.906 - 2.493) | 0.181 | 1.477 (0.834 - 2.617) |
| Poor differentiated  RCC | pT_2_ | 0.245 | 0.52 (0.173 - 1.566) | 0.505 | 0.722 (0.277 - 1.882) | 0.245 | 0.52 (0.173 - 1.566) |
|  | pT_3_ | 0.376 | 1.469 (0.628 - 3.438) | 0.193 | 1.691 (0.767 - 3.724) | 0.376 | 1.469 (0.628 - 3.438) |
|  | pT_4_ | 0.381 | 1.805 (0.482 - 6.756) | 0.378 | 1.784 (0.493 - 6.452) | 0.381 | 1.805 (0.482 - 6.756) |
|  | pN+ | 0.037 | 2.269 (1.05 - 4.905) | 0.029 | 2.262 (1.089 - 4.7) | 0.037 | 2.269 (1.05 - 4.905) |
|  | 5 cm≤tumor size＜10 cm | 0.030 | 3.311 (1.125 - 9.748) | 0.011 | 3.974 (1.37 - 11.527) | 0.030 | 3.311 (1.125 - 9.748) |
|  | Tumor size ≥10 cm | 0.021 | 4.734 (1.265 - 17.72) | 0.035 | 4.01 (1.1 - 14.625) | 0.021 | 4.734 (1.265 - 17.72) |

*reference groups are the same as described in Table 3 and 5

|  | Clinical pathological datas* | OS | | CSS | |
| --- | --- | --- | --- | --- | --- |
|  |  | P value | Hazard ratio (95% CI) | P value | Hazard ratio (95% CI) |
| Advanced  RCC | Preoperative age > 50 | 0.000 | 1.815 (1.565 - 2.104) | 0.004 | 1.273 (1.079 - 1.502) |
|  | pT_2_ | 0.550 | 1.141 (0.741 - 1.757) | 0.698 | 1.112 (0.65 - 1.903) |
|  | pT_3_ | 0.041 | 1.41 (1.014 - 1.963) | 0.169 | 1.35 (0.88 - 2.072) |
|  | pT_4_ | 0.000 | 3.328 (2.369 - 4.677) | 0.000 | 3.373 (2.173 - 5.238) |
|  | pN+ | 0.000 | 2.023 (1.82 - 2.247) | 0.000 | 2.432 (2.137 - 2.768) |
|  | 5 cm≤tumor size＜10 cm | 0.000 | 1.336 (1.213 - 1.471) | 0.000 | 1.816 (1.57 - 2.101) |
|  | Tumor size ≥10 cm | 0.000 | 1.87 (1.674 - 2.088) | 0.000 | 3.151 (2.695 - 3.684) |
|  | Nuclear intermediate differentiation | 0.000 | 1.687 (1.515 - 1.878) | 0.000 | 2.293 (1.954 - 2.692) |
|  | Nuclear poor differentiation | 0.000 | 2.444 (2.179 - 2.741) | 0.000 | 3.792 (3.208 - 4.482) |
|  | Papillary RCC | 0.004 | 1.267 (1.08 - 1.487) | 0.016 | 1.287 (1.049 - 1.579) |
|  | Chromosome RCC | 0.000 | 0.542 (0.395 - 0.743) | 0.000 | 0.448 (0.29 - 0.693) |
|  | Other type | 0.000 | 1.775 (1.624 - 1.94) | 0.000 | 1.489 (1.323 - 1.675) |
| poor differentiation  RCC | Preoperative age > 50 | 0.000 | 1.525 (1.251 - 1.86) | 0.254 | 1.137 (0.912 - 1.418) |
|  | pT_2_ | 0.262 | 1.132 (0.911 - 1.407) | 0.595 | 1.089 (0.796 - 1.489) |
|  | pT_3_ | 0.000 | 1.782 (1.527 - 2.079) | 0.000 | 2.088 (1.665 - 2.618) |
|  | pT_4_ | 0.000 | 4.224 (3.454 - 5.165) | 0.000 | 5.283 (4.011 - 6.96) |
|  | pN+ | 0.000 | 1.83 (1.601 - 2.091) | 0.000 | 2.079 (1.763 - 2.453) |
|  | 5 cm≤tumor size＜10 cm | 0.000 | 1.47 (1.292 - 1.673) | 0.000 | 2.038 (1.685 - 2.466) |
|  | Tumor size ≥10 cm | 0.000 | 2.01 (1.713 - 2.359) | 0.000 | 3.177 (2.553 - 3.953) |
|  | Papillary RCC | 0.864 | 1.027 (0.758 - 1.39) | 0.153 | 0.733 (0.479 - 1.122) |
|  | Chromosome RCC | 0.003 | 0.499 (0.315 - 0.79) | 0.031 | 0.574 (0.347 - 0.949) |
|  | Other type | 0.000 | 1.553 (1.372 - 1.757) | 0.032 | 1.185 (1.015 - 1.382) |

**Table S6. Multivariate analysis of advanced and poor differentiation RCC of SEER database**

*reference groups are the same as described in Table 3 and 5

**Figure. Survival of advanced RCC and poorly differentiated nuclear grade (Fuhrman IV) subgroups.**

**
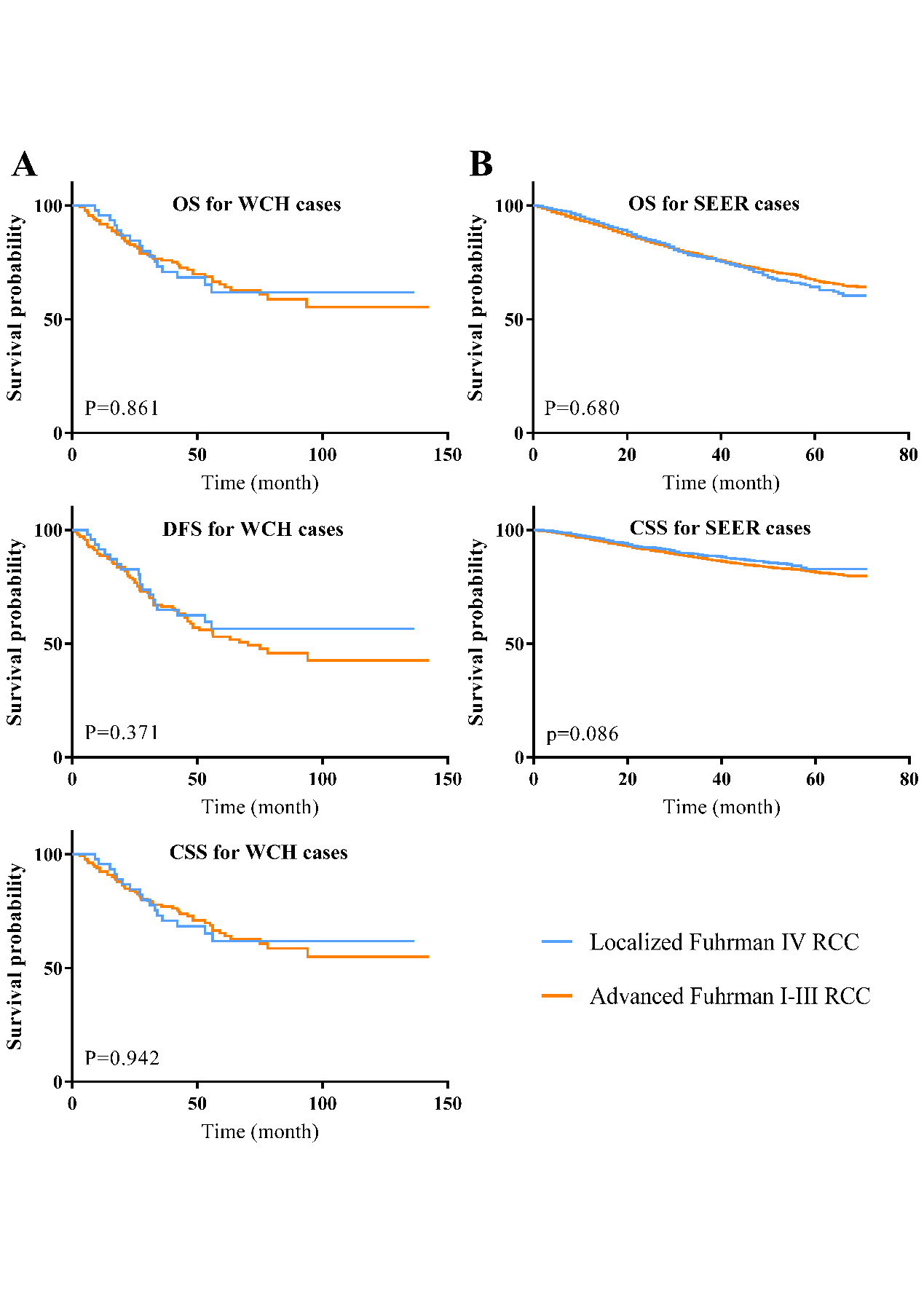
**
